# Response of *Arabidopsis thaliana* and Mizuna Mustard Seeds to Simulated Space Radiation Exposures

**DOI:** 10.3390/life12020144

**Published:** 2022-01-19

**Authors:** Ye Zhang, Jeffrey T. Richards, Alan H. Feiveson, Stephanie E. Richards, Srujana Neelam, Thomas W. Dreschel, Ianik Plante, Megumi Hada, Honglu Wu, Gioia D. Massa, Grace L. Douglas, Howard G. Levine

**Affiliations:** 1 NASA John F. Kennedy Space Center, Kennedy Space Center, Merritt Island, FL 32899, USA; gioia.massa@nasa.gov (G.D.M.); Howard.G.Levine@nasa.gov (H.G.L.); 2 Amentum Services, Inc., LASSO Contract, Kennedy Space Center, Merritt Island, FL 32899, USA; jeffrey.t.richards@nasa.gov (J.T.R.); thomas.w.dreschel@nasa.gov (T.W.D.); 3 NASA Lyndon B. Johnson Space Center, Houston, TX 77058, USA; alan.h.feiveson@nasa.gov (A.H.F.); honglu.wu-1@nasa.gov (H.W.); grace.l.douglas@nasa.gov (G.L.D.); 4 Bionetics Corp., LASSO Contract, Kennedy Space Center, Merritt Island, FL 32899, USA; stephanie.e.richards@nasa.gov; 5 Universities Space Research Association, Kennedy Space Center, Merritt Island, FL 32899, USA; neelamsrjn@gmail.com; 6 KBR, Houston, TX 77058, USA; ianik.plante-1@nasa.gov; 7 Radiation Institute for Science & Engineering, Prairie View A&M University, Prairie View, TX 77446, USA; mehada@pvamu.edu

**Keywords:** space radiation, galactic cosmic rays, solar particle event, *Arabidopsis*, mizuna mustard, seeds

## Abstract

One of the major concerns for long-term exploration missions beyond the Earth’s magnetosphere is consequences from exposures to solar particle event (SPE) protons and galactic cosmic rays (GCR). For long-term crewed Lunar and Mars explorations, the production of fresh food in space will provide both nutritional supplements and psychological benefits to the astronauts. However, the effects of space radiation on plants and plant propagules have not been sufficiently investigated and characterized. In this study, we evaluated the effect of two different compositions of charged particles-simulated GCR, and simulated SPE protons on dry and hydrated seeds of the model plant *Arabidopsis thaliana* and the crop plant Mizuna mustard [*Brassica rapa var. japonica*]. Exposures to charged particles, simulated GCRs (up to 80 cGy) or SPEs (up to 200 cGy), were performed either acutely or at a low dose rate using the NASA Space Radiation Laboratory (NSRL) facility at Brookhaven National Lab (BNL). Control and irradiated seeds were planted in a solid phytogel and grown in a controlled environment. Five to seven days after planting, morphological parameters were measured to evaluate radiation-induced damage in the seedlings. After exposure to single types of charged particles, as well as to simulated GCR, the hydrated *Arabidopsis* seeds showed dose- and quality-dependent responses, with heavier ions causing more severe defects. Seeds exposed to simulated GCR (dry seeds) and SPE (hydrated seeds) had significant, although much less damage than seeds exposed to heavier and higher linear energy transfer (LET) particles. In general, the extent of damage depends on the seed type.

## 1. Introduction

A long-duration mission to Mars will require humans to live in space for up to 2.5 years. The distance of Mars from Earth changes with planetary physics limiting launch opportunities and preventing resupply. Consequently, food may have to be sent years ahead of the crew. Shelf-stable food systems may be five years old by the end of the mission. Under expected ambient storage conditions, critical nutrients and quality factors may degrade over this period of time [1,2]. This possibility creates a critical risk to the provision of adequate food and nutrition to support crew health and performance through such missions. Food crops grown in-mission have the potential to supplement crew nutritional requirements and to act as a psychological countermeasure for the crew by providing a familiar aspect of Earth in the isolation and confinement of deep space [3]. However, seeds and plant growth supplies may also have to be sent ahead of the crew and would need to remain viable for the length of the mission. Deep-space radiation is one of the major factors that could impact the viability of food crops.

Deep-space radiation is composed primarily of protons released from large solar particle events (SPE) and galactic cosmic rays (GCR). GCR consists of approximately 85–90% protons, and 10–13% helium ions (alpha particles), with the remaining 1–2% consisting of high atomic number and energy (HZE) nuclei particles and electrons [4]. For future missions back to the Moon or on to Mars, astronauts and other organisms onboard these deep space missions will be exposed constantly to GCR and occasionally to protons from SPEs. Life on Earth is well protected from these components of deep-space radiation for two reasons: (1) the Earth’s global magnetic field deflects energetic charged particles, and (2) Earth’s atmosphere interacts with them resulting in attenuated energy levels and secondary particles [5]. However, during a mission beyond low Earth orbit (LEO), living organisms will be exposed to more intense deep-space radiation, in addition to secondary particles generated by the shielding materials, the atmosphere, and regolith if near the Lunar or Martian surface.

During a long-term deep space mission, it is expected that crew members in space will be exposed to 1–2 mSv/day dose equivalent rate of GCR and approximately half this amount on planetary surfaces [6,7,8]. Even though the flux levels of GCR particles are very low, GCR contains high linear energy transfer (LET) particles that produce intense ionization as they penetrate through matter. Compared to low LET radiation (e.g., gamma radiation), the estimated average biological effectiveness (or the quality factor) of radiation received on the Martian surface and during transit are 3.05 and 3.82, respectively, primarily due to the Martian atmosphere [6,7,8]. The effective Mars atmospheric shielding is about 21 g/cm^2^, which is much thicker than the spacecraft shielding of the Mars Science Laboratory (MSL)’s Curiosity Rover during its transportation from Earth to Mars [7,8]. For Lunar missions, there is no atmospheric shielding effect. Using data obtained during the MSL mission, it was estimated that a total mission dose equivalent of ~1.01 Sv would be experienced during a round trip Mars surface mission consisting of a 180-day transit (each way) and 500 days on the Martian surface with solar activity similar to that of 2011–2012 [7,8]. For plant seeds, these dose equivalent rates and quality factors may not be applicable because they refer to the biological effectiveness of a given absorbed dose of a specific type of radiation based on human tissue responses. Therefore, in this study, we designed the experiment based on the measurements of the absorbed dose during the MSL mission, which is 400–560 µGy/day during transit and 170–250 µGy/day on the Martian surface at the Gale crater region [7,8].

At the cellular level, radiation induces DNA damage, which to some extent is successfully handled by cellular repair machinery. To counteract DNA damage, a network of cellular pathways, defined as the DNA damage response (DDR) network, mitigates DNA damage by detecting and repairing DNA lesions. These mechanisms consist of cell-cycle regulation, DNA repair, and apoptosis. The basic DDR networks in prokaryotes and eukaryotes are similar, but significant differences exist in the radiosensitivity among different species and the mechanisms that allow access to the lesions by repair enzymes [9]. Plants share many features of chromatin organization and DNA repair with fungi and animals [9,10].

Numerous studies have shown that the effect of ionizing radiation on plants depends upon species, cultivar, development stage, tissue architecture and genome organization, as well as radiation features, e.g., quality, dose, and duration of exposure [11]. Plant seeds have been flown on the ISS and on other venues beyond LEO [12,13,14], however, the radiation environment reported in most of these published studies is not comparable to deep- space radiation, neither in quality nor quantity. Therefore, whether crop seeds, plant growth, and produce quality are significantly affected by long-term exposure to deep-space radiation has yet to be adequately investigated and characterized.

Although current Earth-based radiation facilities can only approximately reproduce the space-radiation environment, recent improvements in technology can be utilized to improve our knowledge about the effect of space radiation on crop plants [15]. This information would in turn address the question of whether fresh food production could provide an important nutritional and psychological contribution on long-duration missions. In this study, two types of plant seeds were included. The first were seeds from *Arabidopsis thaliana,* a model organism that does not have properties that would contribute to a bioregenerative or food capability, but which is currently the most extensively studied plant in spaceflight [16] partially due to the availability of its whole genome sequence and a wide range of genetically modified strains. The second were seeds from an edible mustard plant, Mizuna, *Brassica rapa var. japonica*, which has been identified as a candidate space crop and grown for research and crew consumption on the International Space Station (ISS) [17,18,19,20]. Seeds from both species were irradiated, and subsequently planted and grown to seedlings to demonstrate the impact of the space radiation environment on seeds and their ability to germinate and develop. The range of outcomes between *Arabidopsis* and Mizuna indicates the potential challenges with transferring model organism information to usable systems, and a need to focus research on crops most likely to contribute to spaceflight missions.

## 2. Materials and Methods

### 2.1. Plants

Seeds of wild type *Arabidopsis thaliana* Col-0, and Mizuna (*Brassica rapa var. japonica*), a mustard leafy green, were used for this study. *Arabidopsis thaliana* has been widely used as a model plant organism for microgravity research. *Arabidopsis* seeds were obtained from in-house culture, while Mizuna seeds were purchased from Johnny’s Selected Seeds (Waterville, ME, USA).

### 2.2. Radiation Scenarios

Selected radiation scenarios are summarized in Table 1. Seeds, dry or imbibed, were exposed to protons and titanium ions individually, or using two simulated GCR scenarios (GCR1 or GCR2) and one simulated SPE event. For both GCR scenarios, ions including protons, helium, oxygen, titanium or silicon, and iron were sequentially delivered. GCR1 was a complex mixed radiation field with a similar spectrum of particles identified in GCR. This simulation was designed by Dr. Hada’s team and generated after the above primary beams passed through 20 g/cm^2^ aluminum and high-density polyethylene at 10.345 g/cm^2^ [21]. Under the GCR1 environment, seeds were exposed to a mixture of non-directional secondary particles in which 250 MeV/n protons predominated [21]. The GCR2 scenario was a standard GCR simulation scenario provided by the NSRL physics team for space-radiation risk assessment research without the shielding material [22]. Simulated SPE exposure was achieved using sequential exposures to protons with a broad energy range of 50–150 MeV/n, simulating the August 1972 SPE event [22,23]. Exposures to all radiation scenarios were conducted acutely (within minutes for each single beam, leading to a total of about 20 min. for all particle components) or at a relatively low dose rate (LDR) over a 2–4 h time period (Table 1).

### 2.3. Preparation of Seeds for Irradiation and Growth Study

Mizuna seeds were sanitized by 1 h-hypochlorite fuming (30 mL bleach: 0.5 mL HCL) [24,25], while *Arabidopsis* seeds were sanitized using a standard 70% ethanol method [24]. Sanitized dry seeds for each condition were then transferred to packets prepared from sterile germination paper. For imbibed seed treatments, twenty-four hours before irradiation, 0.9 mL of water was added to each packet to imbibe the seeds, and seed packets with imbibed seeds were then wrapped with several more layers of brown kraft paper sheet to prevent light exposure. All the seed packets, each containing about 25 dry or imbibed seeds, were kept in the dark at approximately 4 °C in a refrigerator, except during the periods of actual or sham (i.e., for controls) radiation exposures when both irradiated and control seed packets were still in the dark, but at room temperature.

Depending on the experimental conditions (Table 1), 26–72 dry or 24 h-imbibed *Arabidopsis* and Mizuna seeds were subject to each radiation exposure treatment, while similar numbers of seeds were retained as non-irradiated controls for comparison with acute or LDR irradiated seeds.

For evaluating germination and early seedling development, two days after irradiation, control and irradiated seeds were planted onto a 0.5% Murishige and Skoog (MS) phytogel media either individually within a pipette tip holder (titanium, proton, and GCR1 experiments) or six to twelve seeds within a 10 cm × 10 cm Petri plate (GCR2 and SPE experiments) at Kennedy Space Center (KSC). Growth conditions approximating those on the ISS (except for CO_2_ concentration) were maintained in a controlled environment chamber (22 °C, 40–45% RH, 150 μmol·m^2^·s^−1^ light, ambient CO_2_ at about 400 ppm, 16 h/8 h photoperiod). Seedlings were harvested at 6–7 days after planting (DAP) for *Arabidopsis*, and 5 DAP for Mizuna for morphological analyses. Images were acquired for evaluating signs of stress and deformation. Seeds were defined as having germinated upon observation of any early sign of germination, including seed coat rupture and radicle emergence. For purposes of assessing viability rates in seeds exposed to Ti-ion exposure, non-germinated seeds as well as germinated seeds without cotyledons, or with radical emergence or showing severely deformed cotyledons without sign of true leaf development were considered non-viable. Imagery analysis for determining cotyledon deformation was conducted only for Ti-ion irradiated *Arabidopsis* seedlings and mizuna experiments. In the titanium-ion irradiation experiment, deformation data were obtained for all seedlings; however, root length measurements for several seedlings were missing due to their roots being broken during the harvest. As a result, the number of plants available for analysis of root length was fewer than the number with deformation data for the titanium-ion experiment. Root length was measured using Rootnav v1.8.1 and Image J software. Root length data are presented in millimeters.

### 2.4. Mathematical Simulation and Radiation Track Analysis

The simulations were conducted with the code RITRACKS [26]. Briefly, a simulation volume is defined, and its base is irradiated by n tracks. The number of tracks is determined from sampling of the Poisson distribution, p(n) = λ^n^ e^−λ^/n!, where λ = ϕA is the average number of tracks, A is the area of the irradiated volume surface, and ϕ is obtained from a given dose D using the well-known equation,

D(Gy) = 1.6 × 10^9^ ϕ(cm^−2^) × LET(keV/μm). (1)

The LET of a particle is calculated using the Bethe’s equation, with corrections [27]. To mimic the contributions of energy deposition from electron tracks of neighboring volumes, periodic boundary conditions (PBCs) were used. Briefly, the electrons that leave the irradiated volume are put back on the opposite side, with the same energy and direction vector [28].

### 2.5. Statistical Analysis

For discrete outcomes, such as viability, germination or deformed cotyledon counts, Fisher’s exact test was used to compare radiation exposure methods (control, acute, or low-dose rate) or to compare doses within each radiation scenario. For comparisons with root length as an outcome, quantile regression was used to compare medians, taking into account the possibility of non-germination and the generally left-skewed distribution of root lengths even if germination had occurred. For defining median root length, non-germination was treated as a length of zero. In other words, a median length of x_0_ is taken to mean 50% of plants had root lengths > x_0_, while the other 50% either did not germinate or if they did, had lengths < x_0_.

## 3. Results

### 3.1. Effect of Titanium Ion and Proton Exposure on Imbibed Arabidopsis Seeds

**Figure 1:**
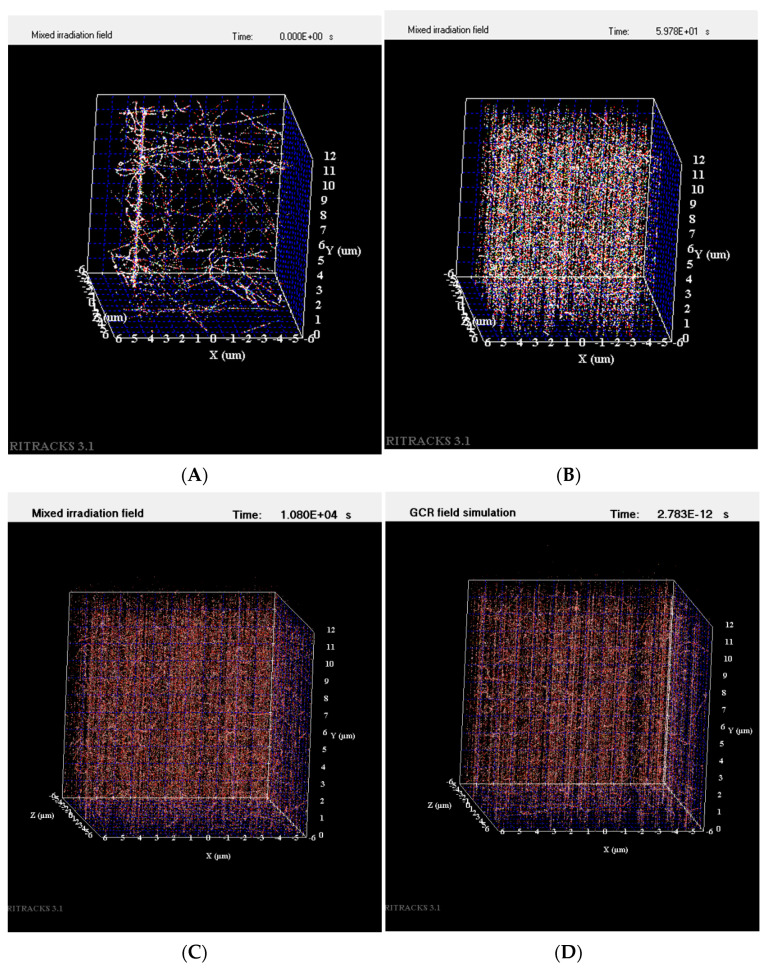
Particle tracks simulated within the volume of one cell. (**A**) A single 300 MeV/n Ti particle track with numerous secondary particles within a cell; (**B**) 250 MeV/n H particle tracks generated within a cell; (**C**) simulated GCR1 mixed irradiation field within a cell; and (**D**) simulated GCR2 irradiation field within a cell.

Irradiation of imbibed *Arabidopsis* seeds was delivered acutely (within minutes), or over a longer duration at a low dose rate (LDR) of 0.166 cGy/min for 40 cGy 300 MeV/n titanium ions (Ti), or 40 cGy 250 MeV/n protons. Mathematical simulations showed that the fluence of these particles over an *Arabidopsis* embryo reached 1750 to 2000 hits/seed for titanium ions (approximately 0.5 hits/cell) and over 800,000 hits/seed for protons (approximately over 200 hits/cell). Examples of simulated Ti, proton, and simulated GCR1 and 2 particle tracks within the volume of one cell (12 μm × 12 μm × 12 μm) are shown in Figure 1.

**Figure 2:**
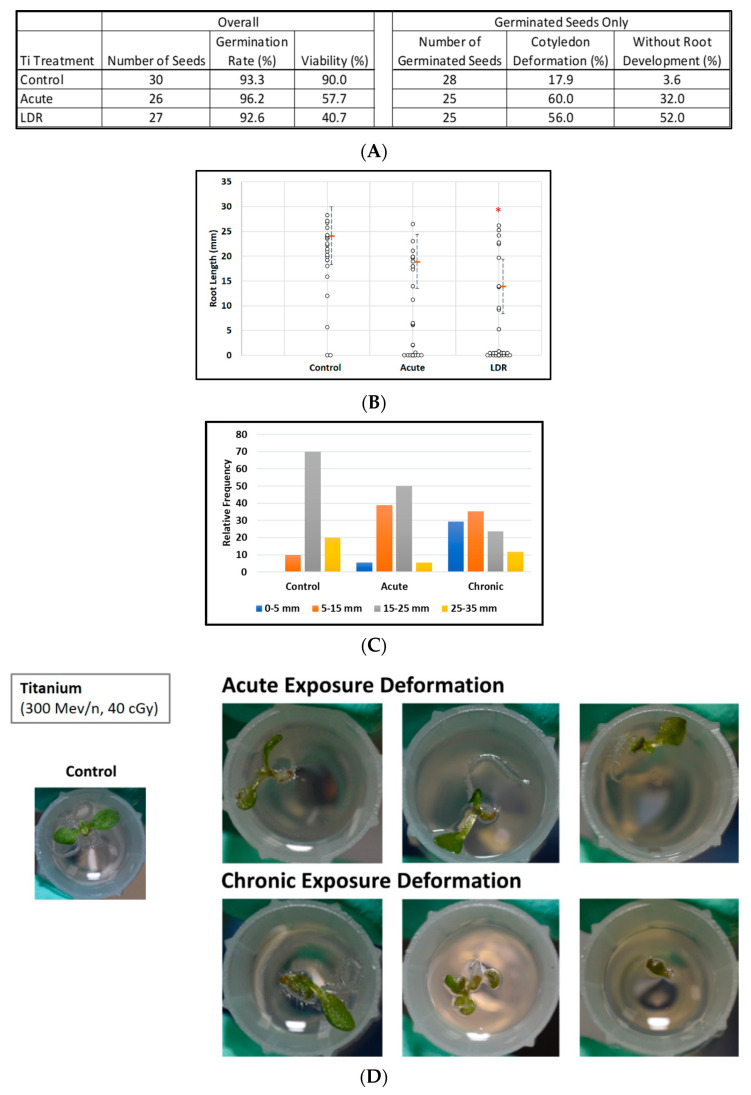
Effect of 300 MeV/n Ti particles on seed germination, viability, and morphological measurements. (**A**) Tables of seeds number with percentages of viability, germination, cotyledon deformation, and absence of measurable roots by experimental group. (**B**) Root length distribution (small circles) in seedlings from control and Ti irradiated seeds. Multiple instances of zero root length are plotted with small jitter so that they can be distinguished. Estimates of 75th percentile root length (orange bars) are shown with 95% confidence limits (dashed lines). (**C**) Comparative histograms of root-length distributions. (**D**) Examples of cotyledon deformation. * Indicates *p* < 0.05 compared to controls.

Exposure to titanium ions had no significant effect on the germination rate, however viability clearly was degraded after irradiation (Figure 2A, left panel). In addition, there were obvious increases in the percentage of cotyledon deformation in the irradiated groups (Figure 2A right panel, Figure 2D). Furthermore (Figure 2A, right panel), exposure to titanium ions had a noticeable effect on root development alone, with no measurable roots in 13 of 25 (52%) germinated seeds (LDR group) as compared with 8 of 25 (32%) seeds (acute group) and only 1 of 28 (3.6%) seeds (control group) (*p* < 0.001, Fisher exact test).

Overall, the distribution of root length in seedlings was shifted downwards (Figure 2C) relative to the control distribution in the two irradiated groups with median lengths of 22.2, 6.4, and 0 mm, for the control, acute, and LDR groups respectively. (The median length was zero for the LDR group because for more than half (14 of 27) of these seeds, germination did not occur or roots did not develop to the point where they were measurable). Even when comparing the upper parts of the root length distribution, we observed a similar pattern; for example, 75th-percentile root lengths were 24, 19, and 14 mm for control, acute, and LDR groups, respectively (Figure 2B). Similarly, when considering only plants with measurable root lengths, estimated median root length ± 1SE was 22.2 ± 2.8 mm for control seeds, decreasing to 17.3 ± 3.0 mm after acute exposure and to 9.5 ± 3.1 mm (LDR exposure).

**Figure 3:**
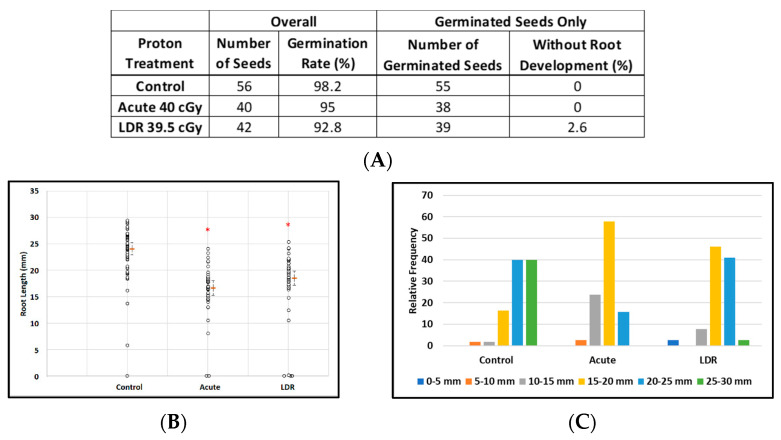
Effect of 250 MeV/n protons on root length in seedlings grown from control and irradiated Arabidopsis seeds. (**A**) *Arabidopsis* seed number and germination rate. (**B**) Root length distribution (small circles) in seedlings from control and irradiated seeds. Multiple instances of zero root length, including non-germinated seeds are plotted with small random jitter so that they can be distinguished. Estimates of median root length (orange bars) are shown with 95% confidence limits (dashed lines). (**C**) Comparative histograms of root-length distributions in plants from germinated seeds. * Indicates *p* < 0.05 compared to controls.

Exposure to protons had virtually no effect on the ability of seeds to germinate with more than 90% germination rates for all experimental conditions: control seeds (98%, 55/56), acute exposure (95%, 38/40), and LDR exposure (92.8%, 39/42) (Figure 3A). However estimated median root lengths differed markedly between the experimental groups: (F(2, 135) = 38.4; *p* < 0.0001). Estimates of median lengths and standard errors were: 24.1 ± 0.6 mm (control), 16.7 ± 0.7 mm (acute exposure), and 18.6 ± 0.6 mm (LDR exposure) (Figure 3B). Figure 3C shows the effects of irradiation on the distribution of root lengths in plants from germinated seeds. Furthermore, exposure to protons, as low as 20 cGy (data not shown), also resulted in significantly shorter and more under-developed roots without obvious deformation of leaf and root tissue. These results suggest that SPEs, which contain protons with a broad energy spectrum, may have a potential impact on plant growth after germination as was also seen for GCR exposures (next section).

### 3.2. Effect of GCR-1 Scenario on Imbibed Arabidopsis Seeds

**Figure 4:**
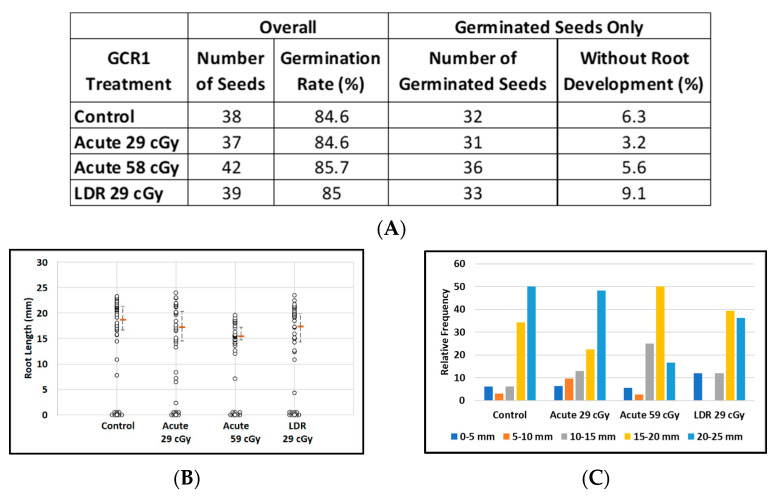
Effect of GCRs on root length in seedlings from control and irradiated *Arabidopsis* seeds. (**A**) *Arabidopsis* seed number and germination rate. (**B**) Root length distribution (small circles) in seedlings from control and Ti irradiated seeds. Multiple instances of zero root length (including non-germinated seeds) are plotted with small random jitter so that they can be distinguished. Estimates of median root length (orange bars) are shown with 95% confidence limits (dashed lines). (**C**) Comparative histograms of root-length distributions in plants from germinated seeds.

About 85% of imbibed *Arabidopsis* seeds germinated in all treatment groups, regardless of exposure to CGR1 radiation (Figure 4A). The relatively large number of non-germinated seeds (six in each group) resulted in a loss of power to discriminate between treatment groups with respect to median root length. Median root lengths were similar for control plants and for plants grown from seeds exposed to simulated acute or LDR GCR1 radiation at 29 cGy, but appeared reduced after exposure to 59 cGy GCR1 radiation (Figure 4B). Considering only the germinated seeds, the median root lengths of plants from seeds exposed to acute 59 cGy GCR1 radiation were noticeably shorter (median length = 15.7 mm as compared with controls, median length = 19.8 mm; *p* = 0.007). The distribution of the root length data from germinated seeds is displayed in Figure 4C.

### 3.3. Effect of GCR2 on Dry Arabidopsis and Mizuna Seeds

**Figure 5:**
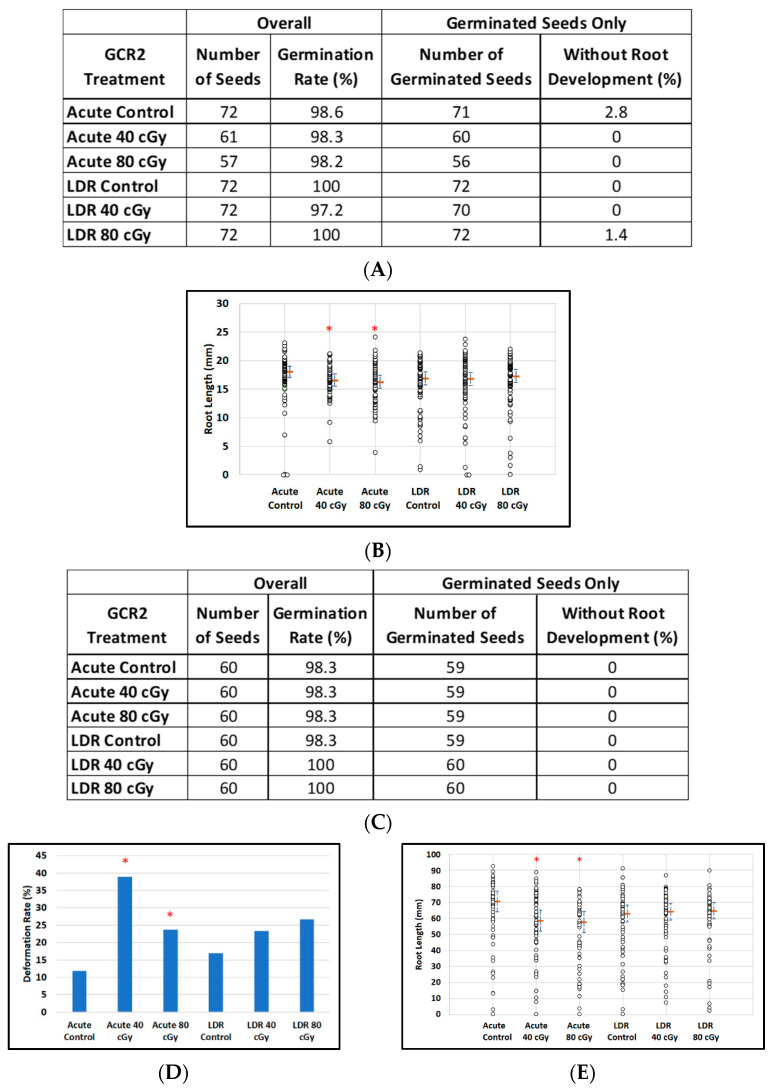
The effect of GCR-2 scenario on root length reduction in seedlings developed from control and irradiated dry seeds. (**A**) *Arabidopsis* seed number and germination rate. (**B**) Root length distribution (small circles) in *Arabidopsis* seedlings from control and irradiated seeds. Estimates of median root length (orange bars) are shown with 95% confidence limits (dashed lines). Multiple instances of zero root length (including non-germinated seeds) are plotted with small jitter so that they can be distinguished. (**C**) Mizuna seed number and germination rate. (**D**) Cotyledon deformation rate in Mizuna seedlings. (**E**) Root length distribution (small circles) in mizuna seedlings from control and irradiated seeds. Estimates of median root length (orange bars) are shown with 95% confidence limits (dashed lines). Multiple instances of zero root length (including non-germinated seeds) are plotted with small jitter so that they can be distinguished. * Shows *p* < 0.05 compared to controls.

Dry *Arabidopsis* and Mizuna seeds were exposed to GCR2 radiation, a simplified and standard six-beam simulation scenario recommended by the NSRL, rather than the GCR1 scenario, which generated a much more complex radiation field for post-irradiation analysis. For *Arabidopsis* seeds, the effect of GCR2 radiation was negligible in that 401 of the 406 seeds planted germinated with notable root development (Figure 5A). For LDR exposure, estimated median root lengths were virtually the same for all dose groups. For acute exposure, there was only a slight indication of a tendency for plants to have degraded root lengths (*p* = 0.04, *p* = 0.02 for doses of 40 and 80 cGy respectively; Figure 5B). In the case of Mizuna, although virtually all seeds (356 of 360) also germinated with root development (Figure 5C), we observed evidence of an acute irradiation effect on the incidence of plants with deformed leaves, with 7 of 59 (11.9%) control plants having deformed leaves (control), as compared with 23 of 59 (39.0%) and 14 of 59 (23.7%) for 40 cGy and 80 cGy exposures (*p* = 0.003, Fisher exact test) (Figure 5D). No such effect was seen after LDR exposure. Similarly, we observed a 17% reduction in median root length of Mizuna plants after acute irradiation compared to the control (95% conf (-27.6%, -7.4%), *p* = 0.001), whereas there was no noticeable effect of LDR irradiation on root length (Figure 5E).

### 3.4. Effect of SPE on Imbibed Arabidopsis and Mizuna

**Figure 6:**
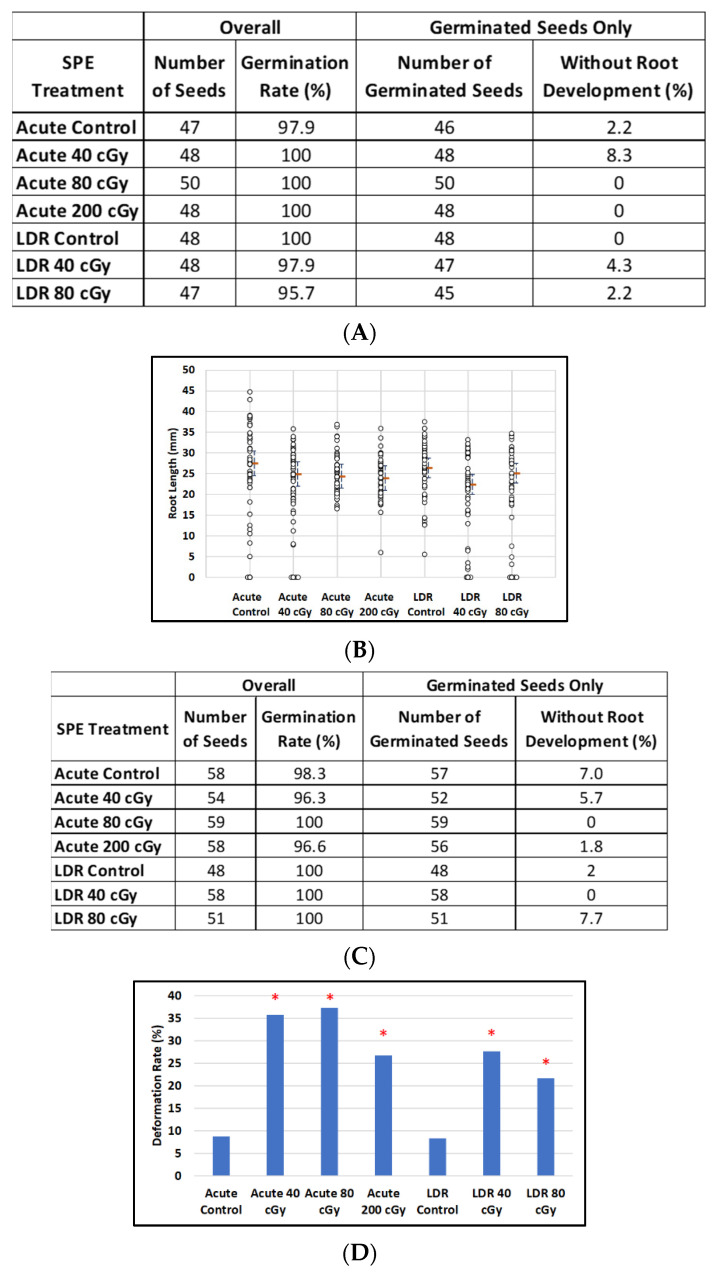

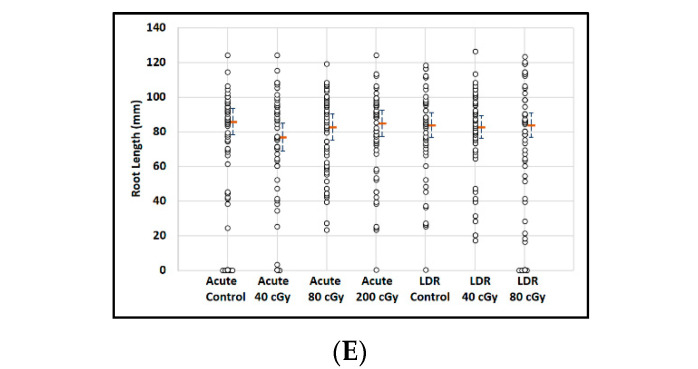
The effect of SPE scenarios on root length reduction in seedlings developed from control and irradiated imbibed seeds. (**A**) *Arabidopsis* seed number and germination rate. (**B**) Root length distribution (small circles) in *Arabidopsis* seedlings from control and irradiated seeds. Estimates of median root length (orange bars) are shown with 95% confidence limits (dashed lines). Multiple instances of zero root length (including non-germinated seeds) are plotted with small jitter so that they can be distinguished. (**C**) Mizuna seed number and germination rate. (**D**) Cotyledon deformation rate in Mizuna seedlings. (**E**) Root length distribution (small circles) in mizuna seedlings from control and irradiated seeds. Multiple instances of zero root length (including non-germinated seeds) are plotted with small jitter so that they can be distinguished. Estimates of median root length (orange bars) are shown with 95% confidence limits (dashed lines). * Indicates *p* < 0.05 compared to controls.

For imbibed *Arabidopsis* seeds, there was no noticeable effect of simulated-SPE dose on percentages of germination) for either acute or LDR exposures (Figure 6A) or on median root length (*p* = 0.35 and *p* = 0.06, comparing all doses, Acute and LDR respectively) (Figure 6B). However, for imbibed Mizuna seeds exposed to acute simulated SPE radiation (Figure 6C), percentages of negative outcomes (non-germinated seeds or plants with deformed cotyledon structure) tended to increase with dose (Fisher’s exact test comparing all doses including control; *p* = 0.0011, Figure 6D). For LDR exposure, percentages of negative outcomes also tended to increase with dose, however the effect was not as striking (Figure 6C, Fisher’s exact test, *p* = 0.034). There was no noticeable effect of dose on median root length for either type of exposure (*p* = 0.40 and *p* = 0.97, comparing all doses, acute and LDR respectively) (Figure 6E).

## 4. Discussion

For future missions back to the Moon or on to Mars, living organisms will be exposed constantly to GCR and occasionally to particles from large solar particle events (SPE), even with effective shielding from the structural materials of vehicles, surface bases, the Martian atmosphere, and the Moon and Mars themselves. Transit vehicles will include protected spaces for astronauts with significantly heavier shielding; however, there are currently no requirements being developed for shielding plant growth or seed storage areas, not only for transit vehicles, but also for Lunar or Martian surface bases. Our study focused on anticipated radiation exposure for long-duration deep space missions such as a 3-year Mars mission. Table 2 summarizes estimates of total GCR dose equivalent rates for different space environments [6,7,29,30,31,32,33,34,35,36]. For comparison purposes and due to the availability of the data, we presented the dose equivalent rates in Table 2 even though these dose equivalent rates were estimated based on human biological effectiveness, which may not be applicable to plants.

For our study, we used 40 cGy to simulate the total GCR dose expected to be experienced during a 3-year Mars mission based on the measurements from the MSL Curiosity Rover Mission during 2011–2012 [7,8]. We also chose a higher dose point of 80 cGy for all simulated GCR irradiation experiments to evaluate the dose response and possible maximum GCR impact on seeds. For some experiments, 20 cGy exposures were used as well. Compared to GCR, the occurrence time, duration, and fluence of an SPE are not predictable. Therefore, for the SPE simulation, we used the model developed by the NSRL based on combining characteristics of two large historical events, namely the fluence of the August 1972 event along with an energy spectrum similar to the March 1989 event [22,23]. We selected three dose points, 40, 80, and 200 cGy, based on the estimated skin doses with 5 g/cm^2^ to 20 g/cm^2^ shielding during the August 1972 SPE [37]. These dose points allow us to determine potential dose response and compare it with the GCR effects. Our study investigated two fundamental questions: (1) whether deep space GCR or SPE protons impacts plant seeds and their growth, and (2) which factors such as species, dose, dose rate, particle type, or some combination of these produce the most detrimental effects. Seeds received either acute exposure within 10–15 min (40–50 mGy/min), or relatively LDR exposures (1.66–3.33 mGy/min and 4.44 mGy/min for GCR and SPE, respectively). Due to facility constraints, the resulting LDR dose for simulated GCR is higher than the dose rate in deep space, but chronic exposure is more relevant in deep-space scenarios than is acute exposure.

### 4.1. Charged Particle Exposure and Hydrated Arabidopsis Seeds

In the first part of this study, we chose hydrated *Arabidopsis* seeds to expose to various single particles (protons and titanium ions), as well as the more complex GCR1 scenario. The GCR1 scenario was developed by Dr. Hada’s group [21] using sequential primary beams and shielding materials to generate a complex mixed radiation field. This was the first time a simulated GCR scenario has been utilized for plant research.

Hydrated seeds contain growing embryos that are presumably more sensitive to radiation damage than dry seeds and mature plants, therefore, we expected the radiation impact, if any, would be shown in these hydrated seeds. In our study, the seeds were hydrated 24 h before irradiation and maintained at 4 °C in the dark. This experimental condition was developed to maintain the seeds in a hydrated, but still relatively dormant stage, to avoid the influence of environmental fluctuations that might interfere with the seeds’ responses to radiation. In particular, temperature can affect both the germination process and DNA repair efficiency. Another factor is the light level that hydrated seeds are exposed to before, during, and after irradiation. Light regimes have been shown to dictate the specific activation and efficiency of some DNA repair pathways such as recombination or photo-repair in various plants [38,39,40]. In this study, we maintained all dry and hydrated samples in the dark to avoid the effects of different light regimes, which may generate significant variations among different samples and experiments.

**Figure 7:**
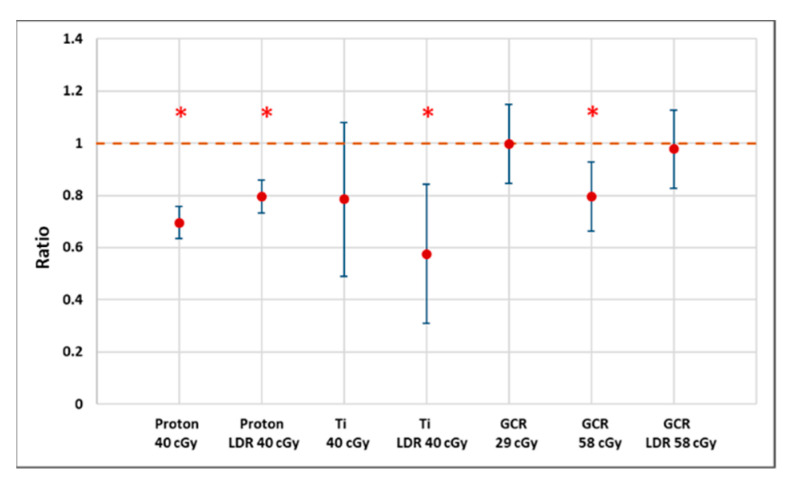
A plot of estimated ratios (treatment to control) of median root length (proton and CGR) or 75th percentiles (Ti) along with 95% confidence intervals showing that the *Arabidopsis* seedlings from irradiated seeds generally have shortened root length. Larger uncertainties for Ti exposure reflect the smaller sample size as well as the failure of many seeds to germinate or produce measurable roots. * Indicates *p* < 0.05 compared to controls.

Exposure of hydrated seeds to these radiation scenarios had little impact on the rate of any signs of germination (including seed coat rupture), which is consistent with other studies. However, morphological changes, especially on root length measurements, of the seedlings cultured from irradiated seeds were found to be dose- and quality-dependent, suggesting that heavier ions cause more severe damage (Figure 7). Because of logistical constraints, we only evaluated seeds subjected to a 24-h imbibition interval. It is possible that at different growing stages with rapidly dividing and differentiating cells of different plant organs, seedlings may show different radiosensitivities [41,42]. The possibility of differential effects of irradiation depending on growth stage needs to be tested further.

### 4.2. Space Mission Relevant Experiments

In actual deep space radiation scenarios, GCR presents a constant extremely low dose rate background radiation field. Because seed imbibition occurs over a relatively short interval (hours to days depending on seed types), maximum cumulative GCR dose during imbibition is expected to be at the milligray range for a 10-day exposure. Therefore, protons released from a large SPE, typically delivered within a couple of days, pose a more significant impact for imbibed seeds than GCR exposure. On the other hand, dry seeds in long-term storage during deep space missions would be exposed to a much higher accumulative GCR dose, which may affect seed viability over long-duration missions. Therefore, we evaluated GCR impact on dry seeds of *Arabidopsis* and Mizuna, and SPE effect on imbibed seeds.

For dry *Arabidopsis* seeds, the GCR-2 scenario did not cause significant changes in germination, viability, or morphology. Root length was impacted more in imbibed seeds by exposure to GCR1 (59 cGy) than in dry seeds by exposure to GCR2 (80 cGy), indicating hydrated seeds are more sensitive to radiation damage as expected. Furthermore, radiation quality plays an important role because 250 MeV/n proton (40 cGy) showed more significant impact on hydrated *Arabidopsis* seeds than simulated SPE (40 cGy) with spectra ranging from 50 to 150 MeV/n, even though both radiation scenarios cause shortened root length. Interestingly, in Mizuna seeds, for the first time, we found a significantly increased abnormality rate in the seedlings developed from both GCR2 and SPE irradiated dry seeds.

In contrast to *Arabidopsis* seeds, dry Mizuna seeds showed an increase in cotyledon deformation with increasing levels of acute GCR2 exposure. This difference between species highlights the need to identify and focus research on crops most likely to contribute to spaceflight missions, as outcomes from model organisms are not universal and may not be applicable. Increased cotyledon deformation was not significant following LDR, indicating potential activation of DNA repair mechanisms. Further evaluation of various crops may help to identify those most resistant to DNA damage and enable identification and screening for the most efficient repair mechanisms.

### 4.3. Comparison with Other Studies

Numerous studies have shown that the effects of ionizing radiation on plants are significantly influenced by species, cultivar, development stage, tissue architecture and genome organization, as well as radiation features, e.g., quality, dose, and duration of exposure [11]. However, very few of these studies used radiation fields that are comparable to deep space radiation in quality and quantity. Plant seeds have been flown on the ISS and beyond LEO spaceflight missions [12,13,14], which are mostly short-duration experiments. There have been several long-duration exposures of seeds to the space environment using different platforms such as the Materials International Space Station Experiment (MISSE) facility, the EXPOSE facility, and the Long Duration Exposure Facility (LDEF). MISSE and EXPOSE are multi-user facilities designed to operate outside of the ISS. The LDEF was deployed to low-Earth orbit (LEO) at approximately 250 nautical miles in 1984 and had orbited the Earth for about 5.7 years. It was retrieved in 1990 at about 178 nautical miles. At present, MISSE and EXPOSE are still operational. The reports of these studies are not quite consistent. In a 27-month EXPOSE-R experiment stationed outside the ISS, analysis of the germination results revealed that the tomato seeds did not survive the exposure to radiation with or without UV. *Arabidopsis* seeds survived only in a dark layer shielded from UV exposure and were able to germinate and further develop [43]. In another report from Tepfer et al. [44], the EXPOSE-R exposure (without UV) significantly reduced the germination rate of seeds from two wild type *Arabidopsis* lines: Columbia (Col-0) and Wassilewskija (Ws-2), with Ws-2 showing more sensitivity to space radiation exposure than Col-0. DNA degradation was found in the seeds exposed to UV, but not significantly in those shielded from UV exposure [44]. In contrast, under a different space environmental condition in the LDEF flight, tomato seeds maintained at a controlled climate of 14 psi with 15% humidity at an altitude between 175 to 275 nautical miles only showed that the pores of the strophiole region of the flight seeds were larger in size than those in the ground control seeds, which might affect germination [12].

For other space radiation related research platforms, in a very recent experiment using the Cosmic Ray Exposure Sequencing Science (CRESS) payload system, a 1U CubeSat design, *Arabidopsis* seeds (under 1 atm) were exposed to the stratosphere (36–40 km) environment above Antarctica in a 30-day, long-duration high altitude balloon mission [45]. In a parallel experiment, dry seeds were exposed to a 40 cGy GCR1 simulation at NSRL. GCR and stratosphere exposed seeds showed significantly reduced germination rates of 76.4% and 82.5%, respectively compared to 98% for the controls. Significantly elevated somatic mutation rates (and developmental aberrations) were also revealed in these GCR or stratosphere exposed seeds with the GCR exposure generating significantly higher mutation rates than that of Antarctica. These mutations also resulted in the death or delayed growth of certain plant organs. Heritable mutations were found in the second generation of the GCR irradiated seeds [45]. DNA damage, degradation, and mutation are the major findings of radiation impacts on seeds and plants in many studies [9,10,11,44,45]. Further investigations are needed to fully characterize DNA damage and DNA damage repair in response to space radiation, as well as the multigenerational effects.

In addition to flight studies, numerous ground radiation studies have been conducted using ground-based radiation sources. Many of these studies did not target space exploration but aimed to evaluate impacts of radiation accidents on the environment, as well as using radiation as a tool to generate desired mutations to benefit agriculture. Among these studies, those conducted in highly contaminated radiation background areas at a dose rate relevant to deep space are particularly of interest. Many studies have been conducted to investigate the effect of chronic low-dose rate exposure on biological organisms including plants. Some have reported significant genotypic and phenotypic changes in plants within the exclusion zone with a relatively low dose-rate [46,47,48].

In general, our findings are in agreement with these studies, and by using ground based GCR and SPE simulations, we provided further evidence that space radiation exposures affect both dry and imbibed seeds.

## 5. Conclusions and Future Work

In our study, we have confirmed for the first-time ground-based simulation of GCR and SPE has an impact on *Arabidopsis* and Mizuna seeds. We observed dependence of outcome measures on dose, dose rate, and radiation quality, with heavier ions causing more severe damage. Exposures to simulated GCR (dry seeds) and SPE (hydrated seeds) had much less effect compared to exposures to heavier and higher LET particles, but there were significant impacts that appear to be dependent on the seed type. As expected, hydrated seeds were more sensitive to radiation exposure than were dry seeds.

Complementary to the results we presented here, it is very important to understand the cellular and molecular basis of these findings. Numerous parameters can be evaluated in future studies to provide deeper insights in the mechanisms behind these reported findings, from analyzing relative sizes and cellular organization of the apical meristem and elongation zone, to conducting multi-omics analyses. Plant DNA damage response and repair mechanisms in response to space radiation also need to be fully characterized. To this end, we are currently conducting an RNAseq analysis using the harvested seedling samples from this study. In addition, we are in the process of evaluating the degree to which GCR and SPE radiation exposures affect the ability of seeds of mizuna, lettuce, and tomato to develop into adult plants and edible fresh produce. 

In space, plant seeds are exposed to multiple space environmental factors, such as altered gravity, suboptimal growth conditions including water stress, high CO_2_ and environmental volatile organic compound (VOC) levels, ethylene generation, and altered air pressure that may cause stress and impact plant growth. Furthermore, plants developed from sanitized seeds may be more susceptible to opportunistic pathogens from the unique microbiome of a transit vehicle. The space radiation environment may further impair the ability of seeds and plants to adapt to these environmental challenges during long-duration missions. In order to support successful integration of fresh food production systems in future spaceflight missions, more studies need to be conducted to understand radiation impacts on a wider variety of space crop candidates and other developmental stages in the plant life cycle, such as young seedling growth, embryogenesis, and seed maturation. More studies are also needed to identify physical or biological methods that efficiently protect or repair seeds from deep space radiation.

## Figures and Tables

**Table 1:** Radiation exposure scenarios at the NASA Space Radiation Lab (NSRL).

*Model Organism*	*Scenario*	*Particle/s*	*Total Primary Dose Delivered (cGy)*	*Dose Rate (cGy/min)*	*Shielding*	*Total Dose Seeds Exposed (cGy)*
Imbibed *Arabidopsis* seeds	Proton Particles	250 MeV/n H^1^+	20	Acute	N/A	20
			40	Acute	N/A	40
			39.5	0.166	N/A	39.5
Imbibed *Arabidopsis* seeds	Titanium Ions	300 MeV/n Ti^48^+	20	Acute	N/A	20
			40	Acute	N/A	40
			39.5	0.166	N/A	39.5
Imbibed *Arabidopsis* seeds	GCR1: Galactic cosmic ray (GCR) simulation	44% 344 MeV/n H^1^+ 19% 344 MeV/n He^4^+ 24% 450 MeV/n O^16^+ 13% 950 MeV/n Fe^56^+	40	Acute	20 g/cm^2^ Aluminum and high density polyethylene 10.345 g/cm^2^	29
				0.166		
			80	Acute		58
				0.166		
Dry *Arabidopsis* seeds and Mizuna seeds	GCR2: Simplified GCR simulation	35% 1000 MeV/n H^1^+ 39% 250 MeV/n H^1^+ 18% 250 MeV/n He^4^+ 6% 350 MeV/n O^16^+ 1% 600 MeV/n Si+ 1% 600 MeV/n Fe^56^+	40	Acute	N/A	40
				0.333		
			80	Acute		80
				0.333		
Imbibed *Arabidopsis* seeds and Mizuna seeds	SPE: 1972 simulation	50–150 MeV/n H^1^+	40	Acute	N/A	40
				0.444		
			80	Acute		80
				0.444		
			200	Acute		200

**Table 2:** GCR dose equivalent rate (mSv/day) at different locations.

Conditions	Mars Mission (Mars Science Lab) [6,7,23]	Lunar Mission (Apollo) [24,25]	On ISS * (~400 km) [29]	Sub-Orbital * (~120 km) [30,31]	Stratosphere above Antarctica * (30–37 km) [32,33]
Transit Journey	1.84 ± 0.33	0.7–3	~0.5 (50–80% from GCR particles)	~0.03–0.336 (Mercury 3)	0.4–0.6
Surface	0.64 ± 0.12 (solar min)	0.24–0.30 (solar max); 0.67–1.04 (Solar min)			

* Total dose equivalent rate, including GCR particles.

## Data Availability

The data will be archived in the NASA Life Sciences Data Archive (LSDA) site.
